# Tissue and host species-specific transcriptional changes in models of experimental visceral leishmaniasis

**DOI:** 10.12688/wellcomeopenres.14867.2

**Published:** 2019-01-02

**Authors:** Helen Ashwin, Karin Seifert, Sarah Forrester, Najmeeyah Brown, Sandy MacDonald, Sally James, Dimitris Lagos, Jon Timmis, Jeremy C Mottram, Simon L. Croft, Paul M. Kaye

**Affiliations:** 1 Centre for Immunology and Infection, University of York, York, YO10 5DD, UK; 2 Department of Immunology and Infection, London School of Hygiene & Tropical Medicine, London, WC1E 7HT, UK; 3 Bioscience Technology Facility, Deptartment of Biology, University of York, York, YO10 5DD, UK; 4 Dept of Electronic Engineering, University of York, York, YO10 5DD, UK

**Keywords:** Leishmania, infection, host response, transcriptomics, immunopathology

## Abstract

**Background**: Human visceral leishmaniasis, caused by infection with *Leishmania donovani* or *L. infantum,* is a potentially fatal disease affecting 50,000-90,000 people yearly in 75 disease endemic countries, with more than 20,000 deaths reported. Experimental models of infection play a major role in understanding parasite biology, host-pathogen interaction, disease pathogenesis, and parasite transmission. In addition, they have an essential role in the identification and pre-clinical evaluation of new drugs and vaccines. However, our understanding of these models remains fragmentary. Although the immune response to *Leishmania donovani* infection in mice has been extensively characterized, transcriptomic analysis capturing the tissue-specific evolution of disease has yet to be reported.

**Methods**: We provide an analysis of the transcriptome of spleen, liver and peripheral blood of BALB/c mice infected with *L. donovani*. Where possible, we compare our data in murine experimental visceral leishmaniasis with transcriptomic data in the public domain obtained from the study of *L. donovani*-infected hamsters and patients with human visceral leishmaniasis. Digitised whole slide images showing the histopathology in spleen and liver are made available via a dedicated website, www.leishpathnet.org.

**Results:** Our analysis confirms marked tissue-specific alterations in the transcriptome of infected mice over time and identifies previously unrecognized parallels and differences between murine, hamster and human responses to infection. We show commonality of interferon-regulated genes whilst confirming a greater activation of type 2 immune pathways in infected hamsters compared to mice. Cytokine genes and genes encoding immune checkpoints were markedly tissue specific and dynamic in their expression, and pathways focused on non-immune cells reflected tissue specific immunopathology. Our data also addresses the value of measuring peripheral blood transcriptomics as a potential window into underlying systemic disease.

**Conclusions:** Our transcriptomic data, coupled with histopathologic analysis of the tissue response, provide an additional resource to underpin future mechanistic studies and to guide clinical research.

## Introduction

Of the many diseases associated with infection by the protozoan parasite *Leishmania*, visceral leishmaniasis (VL) represents one of the most challenging to understand in terms of its pathophysiology. Unlike cutaneous leishmaniasis, where parasite growth and the pathological consequences of the ensuing immune response are largely confined to the site of transmission, VL is characterized by systemic spread of parasites, multi-organ involvement and a systemic response that is fatal without treatment. Systemic cytokine mediated immunosuppression ^1, 2^, T cell functional defects ^3– 7^, alterations to the structural integrity of the lymphoid tissue ^8– 10^ and systemic coagulopathies ^11, 12^ have all been proposed as mechanisms that promote parasite survival, render the host susceptible to concomitant secondary infections and/or act as negative prognostic markers. Evidence from animal models suggests that tissue specific control over immunity and immunopathology also exists, that may in part reflect extremes within the clinical spectrum of VL ^13, 14^. In spite of the broad impact of infection on a range of key physiological processes, a systems-wide appreciation of the underlying immune, metabolic and physiological changes associated with VL has yet to emerge.

Over the past several years, transcriptomic profiling has been adopted as a key methodology for generating an unbiased view of many disease processes. The application of transcriptomic profiling has provided valuable insights into the pathogenesis of many infectious and non-infectious diseases ^15– 26^, and on the response to drugs, vaccines and other immunotherapeutic interventions ^27– 31^. From a clinical perspective, the application of whole blood transcriptomic profiling (WBTP) opened a new era in clinical monitoring of disease, fuelled notably by the work of Chaussebel and others that illustrated its potential to provide insights into systemic disease processes and serve as a clinical tool ^19, 32– 34^. Nevertheless, whether WBTP provides information reflecting a subset of systemic events or is an avatar of that response remains to be fully determined. Indeed, studies formally comparing whole blood with the systemic tissue transcriptome are rare ^21^.

Transcriptomic profiling in leishmaniasis has been limited to date. In human disease, this has largely focused on cutaneous leishmaniasis ^35– 37^. A single study has reported on transcriptional changes in the draining lymph node of Sudanese patients with VL before and after treatment with sodium stibogluconate, identifying a potential role for nuclear factor of activated T cells (NFAT)-regulated immune responses in treatment response ^38^. One study has examined whole blood transcriptional responses in healthy controls versus asymptomatic and symptomatic VL patients in Brazil ^39^. Three studies have evaluated transcriptional changes in the spleen of hamsters infected with *L. donovani* ^40– 42^. Here, we have applied transcriptional profiling to address key aspects of the tissue specific response to *L. donovani* infection in mice. We describe key differences between immune response and metabolic events occurring in the principal target organs of liver and spleen, as well as in blood, and where possible through publically available data, provide a formal comparison between the splenic response in mice and hamsters and between murine and human whole blood.

## Methods

### Ethics statement

Experiments were approved by the Animal Welfare and Ethics Review Bodies of the University of York and the LSHTM and the work was performed under UK Home Office license (PPL 60/4377; PPL 70/6997; PPL 70/8207). Mice were killed by cervical dislocation prior to tissue collection, as described below.

### Mice and infections

6–8-week-old female BALB/c mice (Charles River, Margate, UK) weighing 20±1 gm and health screened to FELASA 67M standard and maintained under specific pathogen free conditions in individually ventilated cages were used in this study. *Leishmania donovani* (LV9) parasites were maintained in B6. *Rag1* ^-/-^ mice and amastigotes prepared following tissue disruption, cell lysis and differential centrifugation, as described elsewhere ^43^. 2×10 ^7^ amastigotes in 150 μl RPMI were injected intravenously (i.v.) via the lateral tail vein and without anaesthetic to initiate infection. After infection, mice were allocated to cages of 5 and provided food and water *ad libitum*. Experiments reported here are derived from two cohorts of mice (designated CRACK-IT_1 and CRACK-IT_2; 20 mice per cohort, n=5 per infection time point and for time-matched uninfected controls). In CRACK-IT_1, mice (n=5) were killed by cervical dislocation at day 15, 17 and 21 post infection. Control naïve mice were killed on the equivalent of day 14 p.i. for logistical reasons. In CRACK-IT_2, infected mice (n=5 per group) were killed at day 36 and 42, alongside a control naïve group. All animals were killed and processed over an approximate time period of 4–6h beginning in the morning. Tissues were aseptically removed post mortem and stored/processed as detailed below. All downstream tissue analysis was performed blinded to group by investigators not involved in animal handling.

### Histology and quantitative morphometry

Spleens and livers were embedded in OCT, snap frozen and stored at -80°, before preparing 7 µm cryosections. All subsequent steps were carried out at room temperature. For gross morphology, sections were fixed for 5 minutes in ice-cold acetone and rehydrated by immersion for 2–3 minutes in 90% ethanol, 70% ethanol and finally ddH _2_O. Sections were transferred to Harris’s haematoxylin (Sigma-Aldrich, UK) for 5 minutes, then ‘blued’ in running tap water for 5 minutes. Slides were differentiated using 1% acid alcohol for 5 seconds, washed in tap water for 3minutes and stained with 1% w/v eosin (Sigma-Aldrich, UK) in distilled water for 3 minutes. Slides were washed for 3 minutes in ddH _2_O and dehydrated through alcohols, cleared in Xylene and mounted in DPX.

For immunohistochemistry, sections were fixed for 5 minutes in ice cold acetone and rehydrated for 2 x 5 minutes in wash buffer (0.05% w/v BSA in PBS) and blocked for 30 minutes in dilution buffer (5% v/v goat serum in wash buffer). After washing, sections were stained for 45 min with AlexaFlour 647-F4/80 or isotype control (BioLegend, USA; 1:500), counterstained with DAPI and mounted in Prolong Gold. Sections were examined using a Zeiss Axio Scan Z1 with a x 20 objective. Granuloma size and distribution was quantified using image analysis software TissueQuest 4.0 (TissueGnostics, Vienna, Austria), blind to treatment group. DAPI was used as the master channel to identify all events within the regions of interest, and clustering of F4/80 ^+^ cells was used to identify granulomas. All granulomas within a 1mm ^2^ region of interest were identified as individual sub regions of interest, and size and cell density was recorded.

### Tissue transcriptomics

RNA was isolated from tissue/blood samples and amplified via Agilent low-input Quick Amp labelling kit (Agilent Technologies). Amplified RNA was then assayed with Agilent SurePrint G3 mouse GE 8x60k microarray chips that were scanned with an Agilent C Scanner with Surescan High Resolution Technology (Agilent Technologies). The data were normalized using the percentile shift method to the 75 ^th^ percentile. Identification of differentially expressed (DE) genes between infected and naïve samples was performed using the Benjamini and Hochberg false discovery rate (FDR) correction for pairwise comparisons of infected vs. naïve samples ^44^. Where multiple probes for a single gene were present, the average expression level was used. This analysis was performed with GeneSpring software (version 9; Agilent) as a standard 5% FDR, with the variances assessed by the software for each t test performed. A 2-fold expression criteria (Log2 FC = 1)was then applied to each gene list. For DE analysis in CRACK-IT_1, a single group of naïve mice were killed at d14 for comparison with samples of infected mice taken at d15, d17 and d21 p.i. For CRACK-IT_2, DE analysis was performed using matched naïve samples for each tissue taken at each time point (see Results).

Gene ontology analysis was performed using GeneSpring (Agilent), and pathway analysis was conducted using Ingenuity Pathway Analysis software (Ingenuity Systems). For the identification of upstream regulators in IPA, we used the z score, which reflects the extent to which known target genes are regulated in the expected direction, whereby positive z scores predict activation of the regulator whereas negative z scores predict inhibition of the regulator. z scores of >2 and <-2 are considered significant. Gene set enrichment analysis (GSEA) ^34^ was performed to identify enriched gene sets associated with each phenotype (i.e. infected at each time point vs. naïve). Data were collapsed to gene set against an Agilent mouse array chip file (~24K genes) and parameters set for GSEA were: permutations =1000; permutations type = gene set (sample n<7) or phenotype (sample n>7). Putative protein-protein interactions were identified using STRING ^45^. Interferon-inducible genes were identified using Interferome v2.0 ^46^ Non-weighted Venn diagrams were generated using Venny 2.1 ^47^. Hamster transcriptomic datasets were from 41. Comparisons were made based on ENSEMBL ids and gene symbol and annotation was used to compare hamster / mouse orthologs. Human transcriptomic data sets were from 39. Weighted Venn diagrams were generated in R using the eulerr package ( https://cran.r-project.org/package=eulerr) and correlation plots were created using the ggplot2 package ( https://cran.r-project.org/web/packages/ggplot2/index.html).

## Results

### Splenic response to *L. donovani* infection in BALB/c mice

**Figure 1.**
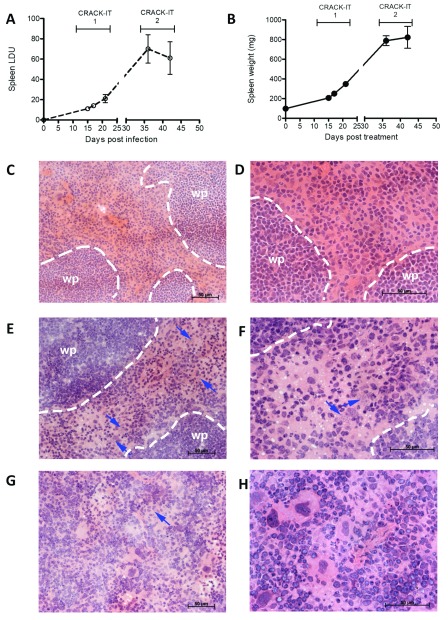
Splenic response to *L. donovani* infection. Spleens from BALB/c mice infected with *L. donovani* were removed at 15-, 17-, 21-, 36- and 42-days post infection, along with spleens from naïve age matched controls. **A**. Parasite burdens were determined by impression smears and are represented as mean LDU ± SE (n=5 mice per time point). **B**. Spleen weight for naïve and infected mice (mg ± SE). **C**– **H**. Representative histology of spleens from naïve mice ( **C** and **D**) and from d17 ( **E** and **F**) and d42 ( **G** and **H**) infected mice. Hematoxylin and Eosin x20 ( **C**, **E** and **G**) and x40 ( **D**, **F** and **H**); scale bars 50 µm. Blue arrows highlight parasite clusters. White dashed lines indicate red pulp (rp) / white pulp (wp) boundary. **G** and **H** show increased frequency of megakaryocytes, indicative of extramedullary haematopoiesis and loss of white pulp / red pulp discrimination. Data are pooled from two independent experiments covering the early and late phase of infection.

**Figure 2.**
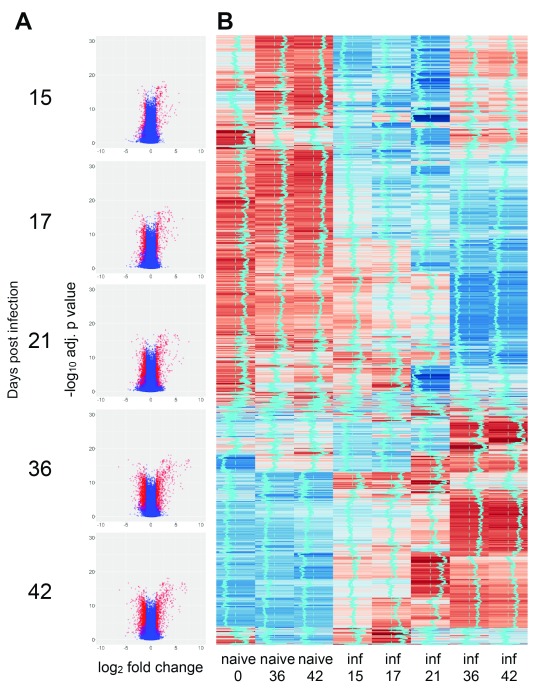
Transcriptomic profiling of spleen response to *L. donovani* infection in BALB/c mice. **A**. Volcano plots showing DE probes at each time point of infection (relative to matched naïve control mice). Data are shown as Log2 FC in expression against Log10 adjusted p value. **B**. Heat map of probe expression intensity across time series. Data are pooled for n=5 mice per group for clarity. Vertical blue trace indicates mean intensity signal. Sample “naïve 0” was used to calculate DE genes for days 15, 17 and 21 p.i. Samples “naïve 36” and “naïve 42” were used to calculate DE genes for their respective time points p.i.

As part of a larger program of research aimed at evaluating multiple aspects of EVL (the CRACK IT VIDR project; see Discussion), we infected two cohorts of female BALB/c mice with 2×10 ^7 ^amastigotes of *L. donovani* (CRACK-IT_1 and CRACK-IT_2) and at defined time points corresponding to increased parasite load and architectural changes in the splenic microenvironment ( Figure 1), spleen tissues were processed for genome-wide transcriptomic profiling by microarray. An overview of the expression data is presented as volcano plots and heat maps for naïve mice (total n=15) and infected mice (total n=25) examined at each time post infection (p.i.) ( Figure 2A and B). Some minor changes were noted in groups of naïve mice examined at each time point, despite confirmation of comparability in age, sex, weight and health status. To account for these minor variations, DE genes were identified by reference to time- and experiment-matched naïve controls ( Figure 2B). Using a mean FDR adjusted p value cut-off of 0.05 and a FC of 2 (Log _2_FC = 1) for at least one of the infection time points, 9634 probes were scored as DE across the entire time series ( Table S1). The number of DE probes increased over time, peaking at d36 p.i. (d15: 1997 probes, Log _2_FC -3.52 to +5.82; d17: 2826 probes, log _2_FC -4.01 to +6.37; d21: 5124 probes, Log _2_FC -4.54 to +7.59; d36: 5051 probes, Log _2_FC -6.45 to +8.36; d42: 4946 probes, Log _2_FC -6.30 to +7.13).

**Figure 3.**
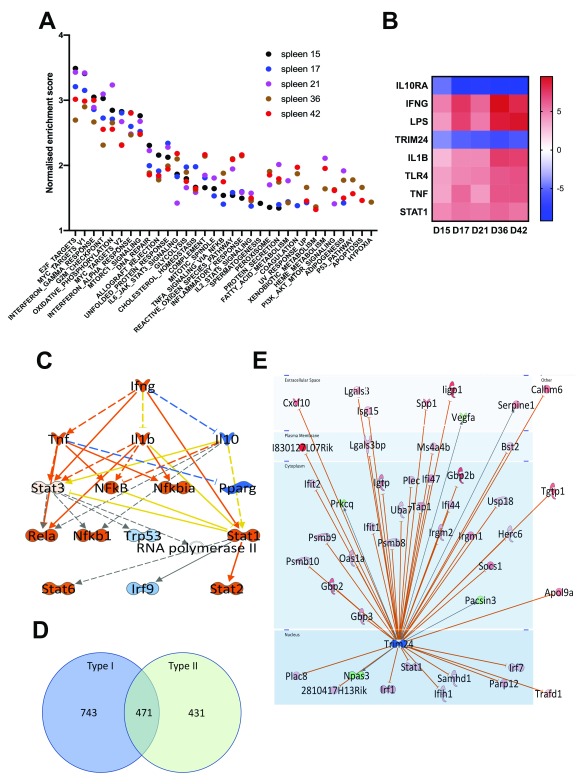
Characteristics of the spleen transcriptomic response to *L. donovani* infection in BALB/c mice. **A**. Data were subjected to GSEA with reference to MSigDB Hallmark gene sets and normalized enrichment score over time is shown for the top 25 gene sets at any given time point. Days post infection are indicated by coloured dots (d15, black; d17, blue; d21, magenta; d36, brown, d42 red). **B**. IPA-predicted upstream regulators presented as a heat map based on z scores ranging from -9 (negative regulators) to +9 (positive regulators). **C**. IPA mechanistic pathway analysis indicating the 16 linked regulatory proteins, that together are predicted to regulate ~14% of all DE genes in the *L. donovani* infected spleen. Predicted relationships are indicated by lines (orange, leading to activation; blue, leading to inhibition; yellow, inconsistent; grey, not predicted). Box shading represents degree of predicted activation (orange) or inhibition (blue). **D**. Venn diagram showing distribution of 1645 Type I and II Interferon-induced genes, as identified using Interferome v2.01. **E**. Predicted Trim24 targets identified in IPA that show upregulated expression during infection. Predicted relationships are indicated by lines: Orange represents leading to activation; grey represents not predicted. Gene symbol box shading reflects extent of upregulation (red) and downregulation (green) in dataset. For an explanation of molecule shapes and relationship types, see http://qiagen.force.com/KnowledgeBase/articles/Basic_Technical_Q_A/Legend.

After removal of multiple probes, 5096 annotated genes were identified within this DE probe set across all time points (d15, 1078 genes; d17, 1653 genes; d21, 2880 genes; d36, 2708; d42, 2805). To determine if genes related to specific biological responses were differentially regulated over time of infection, we used GSEA ^34^. As shown in Figure 3A, Table 1 and Table S2, the most commonly enriched gene sets across the time course were interferon gamma signalling, TNF signalling, myogenesis, allograft rejection, IL-6 – JAK/STAT signalling, angiogenesis, G2M checkpoint, inflammatory response, oestrogen response, epithelial-mesenchymal transition, E2F targets, KRAS signalling and complement. Notably, the breadth of enriched pathways evolved over time. For example, pathways representing various metabolic processes, coagulation, apoptosis and hypoxia are more highly enriched late in infection ( Figure 3A). These changes occurred concurrently with loss of red pulp/white pulp differentiation and the onset of extramedullary haematopoiesis (as evident from the abundance of splenic megakaryocytes; Figure 1G and H).

**Table 1.** Summary of enriched gene sets in the spleen of *L. donovani*-infected BALB/c mice.

Data was subjected to GSEA against the MSigDB Hallmark gene set and represents a summary of the top 10 gene sets (by FDR) for each time point. For full dataset including gene lists, see S2Table. Normalised enrichment scores for all gene sets listed for all time points are shown in Figure 3A.	day 15	day 17	day 21	day 36	day 42
Gene Set Name	FDR q-value				
HALLMARK_KRAS_SIGNALING_UP	7.80E-09	3.07E-11		2.70E-24	1.63E-20
HALLMARK_KRAS_SIGNALING_DN	2.67E-08				
HALLMARK_ALLOGRAFT_REJECTION	1.15E-07	8.95E-10		9.97E-27	
HALLMARK_IL6_JAK_STAT3_SIGNALING	2.48E-07				
HALLMARK_INTERFERON_GAMMA_RESPONSE	2.40E-06	7.24E-26	1.07E-22	2.75E-40	1.88E-27
HALLMARK_ESTROGEN_RESPONSE_EARLY	9.16E-06				
HALLMARK_INFLAMMATORY_RESPONSE	9.16E-06	3.07E-11	1.13E-19	4.08E-25	8.69E-23
HALLMARK_ANGIOGENESIS	1.06E-05				
HALLMARK_COAGULATION	1.77E-05			1.90E-23	
HALLMARK_EPITHELIAL_MESENCHYMAL_TRANSITION	3.20E-05	4.76E-13	1.13E-19	1.52E-18	3.09E-21
HALLMARK_INTERFERON_ALPHA_RESPONSE		1.26E-12			
HALLMARK_HYPOXIA		1.26E-12		3.81E-20	3.38E-25
HALLMARK_MYOGENESIS		1.26E-12			
HALLMARK_G2M_CHECKPOINT		3.07E-11	1.50E-36		1.96E-24
HALLMARK_TNFA_SIGNALING_VIA_NFKB		4.32E-09		2.37E-30	6.38E-26
HALLMARK_E2F_TARGETS			1.50E-36		1.63E-20
HALLMARK_MITOTIC_SPINDLE			1.13E-19		
HALLMARK_IL2_STAT5_SIGNALING			6.16E-19	1.18E-22	
HALLMARK_UV_RESPONSE_DN			2.57E-17		
HALLMARK_XENOBIOTIC_METABOLISM			1.54E-14		
HALLMARK_COMPLEMENT				4.08E-25	9.69E-20
HALLMARK_ESTROGEN_RESPONSE_LATE			1.54E-14		5.18E-22

We next used pathway analysis to look in more detail at the evolution of and the main upstream regulators of the DE genes identified at each time point post infection. Comparative analysis within Ingenuity Pathway Analysis (IPA) indicated that IFN *γ*, LPS, IL-1β, TLR4, TNF and STAT1 were amongst the highest scoring and significant upstream regulators predicted to be activated during infection (with activation z scores ranging from 2.89 to 9.5 and p values from ~10 ^-5^ to 10 ^-48^), whereas IL-10RA and TRIM24 were upstream regulators that were predicted to be down regulated (z scores of ~-4 to -9 and p values from 10 ^-7^ to 10 ^-35^; Figure 3B). It should be noted that predictions made by IPA are based on the expression patterns of genes that are known to be regulated by different upstream regulators and do not require that the upstream regulator itself appears as DE in the data set. For example, at day 36 p.i., activation of IFNγ was predicted by the changes in expression seen in 301 known IFNγ target genes, consistent in this case with an observed Log2FC change in expression of IFNγ of 3.474. IFNγ was further predicted to interact with 694 genes (or 13.7%) of all DE genes via a mechanistic network involving 15 other regulators ( Figure 3C). Similarly, using the Interferome database which contains expression data related to ~4000 interferon regulated genes (v2.01; ^46^), 32.3% (1645/5096) of all DE genes scored as interferon inducible, of which 743 and 431 scored as uniquely regulated by Type I and II (IFNγ) interferons, respectively ( Figure 3D). In contrast, IL-10RA and TRIM24 were predicted to be inhibited based on the expression change of 108 and 41 genes, respectively, although in this case, neither of these predicted upstream regulators was itself DE. The 41 genes that predict inactivation of TRIM24 during *L. donovani* infection are shown graphically according to subcellular localisation in Figure 3E,. The lack of differential expression of IL-10RA and TRIM24 may reflect that function is not transcriptional regulated or that transcriptional regulation was below the Log2FC cutoff employed in this analysis (due to low abundance of cells expressing the gene of interest or only minor changes to mRNA abundance).

**Figure 4.**
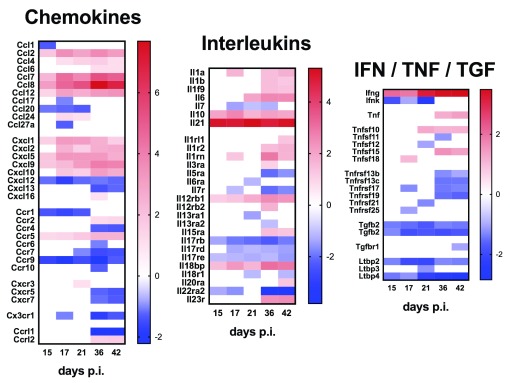
Cytokine and chemokine ligand and receptor gene expression in the spleen of mice infected with *L. donovani.* **A**– **C**. Heat maps representing Log2 fold change in mRNA abundance for chemokines, interleukins and IFNγ / TNF family members and their receptors are shown. Only genes that were DE for at least one time point post infection are shown. Blue blocks represent down-regulated genes and red blocks indicate up-regulated genes. White blocks represent genes that were not significantly DE at a given time point.

Finally, we examined expression of cytokine and chemokine genes as well as genes for their respective receptors, to provide a high-level view of immune pathways activated during infection in this target tissue ( Figure 4). Cytokines with increased mRNA accumulation at all time points included *Il10* and *Il21*, whereas for others (e.g. *Il1b* and *Il6*) there was progressive increase in mRNA accumulation over time. *Ifnγ* mRNA abundance also increased over time post infection, whilst enhanced accumulation of *Tnf* mRNA was observed only late in infection and other TNF superfamily members showed variable responses. Distinct temporal regulation of chemokine mRNA abundance was also observed. In contrast to chemokine ligands, mRNAs for many chemokine receptors were found at lower abundance in infected mice compared to controls (e.g. *Ccr1* and *Ccr9*), and the same trend was also observed for some cytokine receptors, notably *Il17rb*, *Il17rd* and *Il17re*.

### Comparative analysis of splenic response in mice and hamsters infected with *L. donovani*

**Figure 5.**
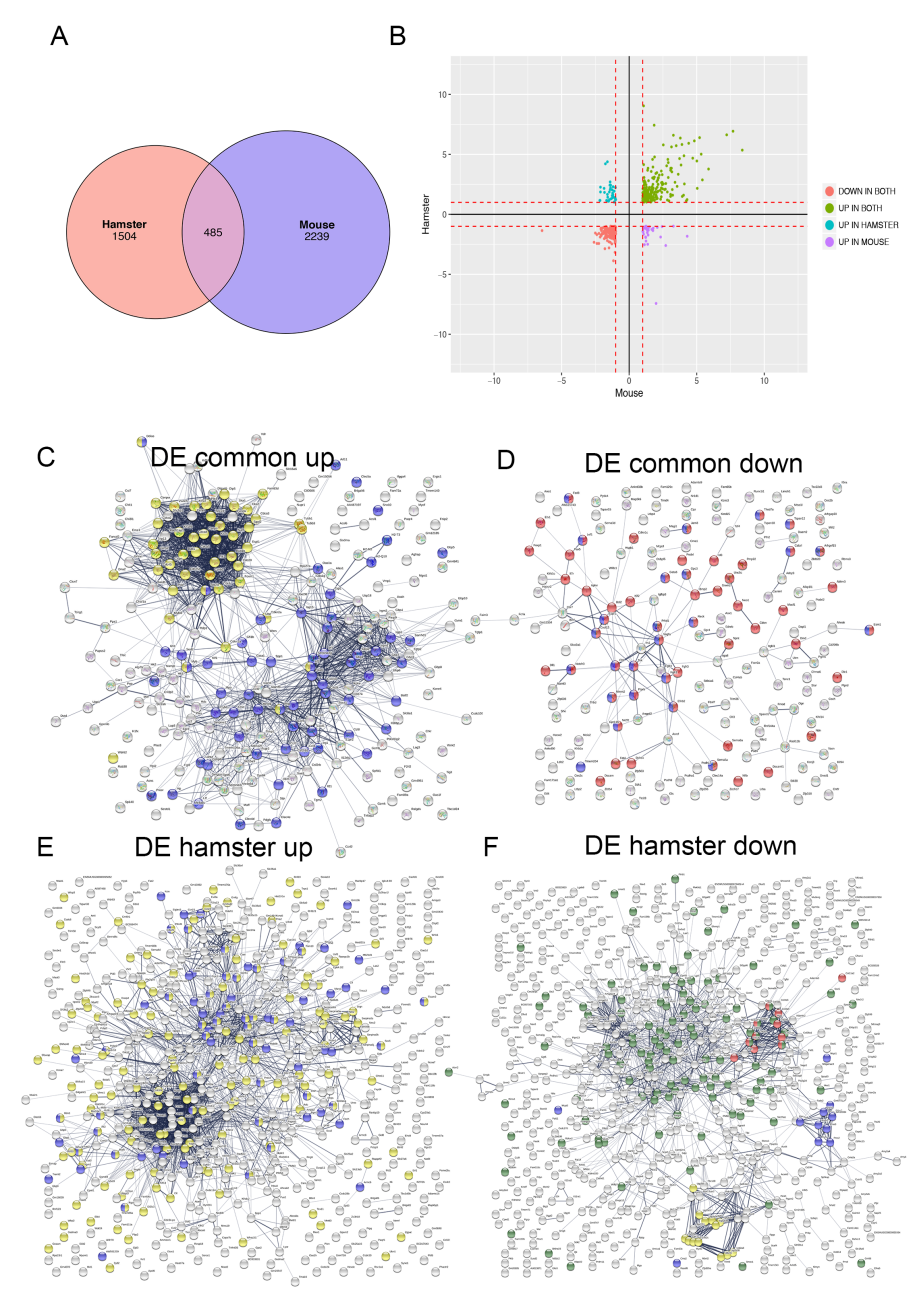
Comparison between DE genes in hamster and mouse spleen. DE genes identified in mouse spleen (d36 p.i.; this study) were compared to those identified in 41. **A**. Venn diagram showing overlap of DE genes. **B**. Correlation plot of Log2FC for mouse and hamster DE genes. **C**– **F**. STRING analysis of predicted protein interactions for DE genes identified as upregulated in both mouse and hamster ( **C**; yellow = cell cycle, blue = immune response), down-regulated in mouse and hamster ( **D**; red = anatomical structure, blue = vascular development), up-regulated in hamster only ( **E**; yellow = negative regulation, blue = immune system), and down-regulated in hamster only ( **F**; green = regulation of multicellular processes, red = complex of collagen fibres, yellow = drug metabolism, blue = serine proteases trypsin domain). All DE genes are shown on the images to provide a visual representation of the extent to which these genes are clustered into common pathways. For full details of all DE genes, see Table S3.

In contrast to murine EVL, hamsters infected with *L. donovani* develop progressive and fatal visceral leishmaniasis, providing a better model of end stage human disease. The heightened sensitivity of hamsters to *L. donovani* infection has been attributed, in part, to a deficiency in NOS2 production, resulting from a promoter polymorphism similar to that seen in humans ^48^. In addition, alterations in immune response associated with T cell exhaustion and myeloid cell dysregulation have been reported based on transcriptomic as well as functional analysis ^41, 42^. However, a detailed comparison of the pathophysiology in infected hamsters and mice has not been conducted. We made use of recently published RNA-Seq data derived from the spleens of hamsters infected with *L. donovani* infection ^41^ to compare these two infection models, albeit with the caveat that different technologies were used for measuring mRNA abundance. Kong *et al.* ^41^ observed 4360 DE transcripts at d28 post infection, of which 2340 (53.7%) were up-regulated and 2020 (46.3%) were down-regulated compared to spleens from naïve hamsters. In comparison, in *L. donovani* infected mice at the peak of splenic infection in our study (d36 p.i.), we identified 41.2% of DE probes as upregulated and 58.8% as down-regulated ( Table S1). By filtering on FDR and FC (<0.05; >2FC), removing the bottom quartile of hamster genes that were expressed with a cpm of <1 and by averaging across multiple probes/reads, we generated three lists of genes, those DE in hamster and not mouse (1504 genes), DE in mouse and not hamster (2239 genes) and those DE in both (485 genes, Figure 5A and Table S3).

Amongst common DE genes, there were those that were up or down consistently in both species, and in more limited number, some that showed differential expression in alternate directions (35 up in mouse and down in hamster and 37 up in hamster and down in mouse; Figure 5B and Table S3). To further examine these DE gene lists, we used STRING, a protein-protein interaction database ^45^. We identified no discernible functional pathways associated with the small number of inversely DE genes. Analysis of common upregulated DE genes identified a highly significant enrichment for immune system process (GO:002376; FDR 7.37×10 ^-23^) and cell cycle process (GO:0022402; FDR 2.75×10 ^-19^) related genes that form two major clusters amongst all commonly upregulated genes ( Figure 5C). Amongst common down-regulated DE genes, although there was no major clustering of genes there was a significant enrichment in GO terms related to changes in morphology, pathways for anatomical structure morphogenesis (GO:0009653; FDR 3.22×10 ^-14^) and vascular development (GO:0001944; 1.82×10 ^-11^), the latter including negative regulators of angiogenesis such as *Mmrn2* and promoters of angiogenesis such as *Vegfa* ( Figure 5D). These data are consistent with the notion that the well documented changes in vasculature associated with murine infection ^9^ also occur during hamster infection.

Amongst DE genes upregulated only in infected hamsters, GO pathways related to negative regulation (e.g. GO: 0048519; FDR 1.13×10 ^-7^) and immune system processes (e.g. GO:0002376; FDR 2.1×10 ^-7^) predominated, again forming significant clustering in the dataset ( Figure 5E). Most notable amongst the top 25 up regulated genes were *Ido1*, a potent negative regulator of immune responses (245-fold upregulated) and *Arg1*, associated with Th2-associated cytokines (36-fold upregulated). Surprisingly, *Il10* was not upregulated in splenic tissue. In contrast, DE genes downregulated in hamsters only related to morphogenesis and organismal processes (e.g. GO:0051239: FDR 5.78×10 ^-9^), complexes of collagen trimers (GO:0098644; FDR 3.4×10 ^-11^), drug metabolism (KEGG:00983; FDR 7.94×10 ^-8^) and serine proteases, trypsin domain (IPR001254; FDR 0.00741). Of the top 25 down-regulated genes, 10 encoded amylase or amylase precursor proteins and 10 have serine peptidase activity ( Table S3).

Amongst DE genes upregulated only in murine infection, genes associated with GO terms related to host defence and immune response again predominated (e.g. GO:0006955; FDR 2.03×10 ^-10^; GO:0006952; FDR 4.96×10 ^-10^). Notable amongst the top 25 upregulated genes were *Ptgs2* (or Cox-2), a key enzyme in the pathway leading to prostaglandin synthesis (124-fold upregulated), *Tff1*, associated with chronic inflammation (75-fold upregulated), *Ctsg*, thought to promote excessive inflammation (33-fold upregulated) and the neutrophil related proteins *Lcn2* and *Ngp* (19.7 and 24.3-fold upregulated respectively). Notable in the top 25 downregulated genes were *Cd27* (langerin; 23-fold downregulated), the sodium voltage gated channel encoding *Scn3a* (18-fold down regulated), *Cd209c* (SIGNR2; 9-fold), and *Il22ra2*, a soluble receptor that inhibits IL-22 activity (12.7-fold).

**Figure 6.**
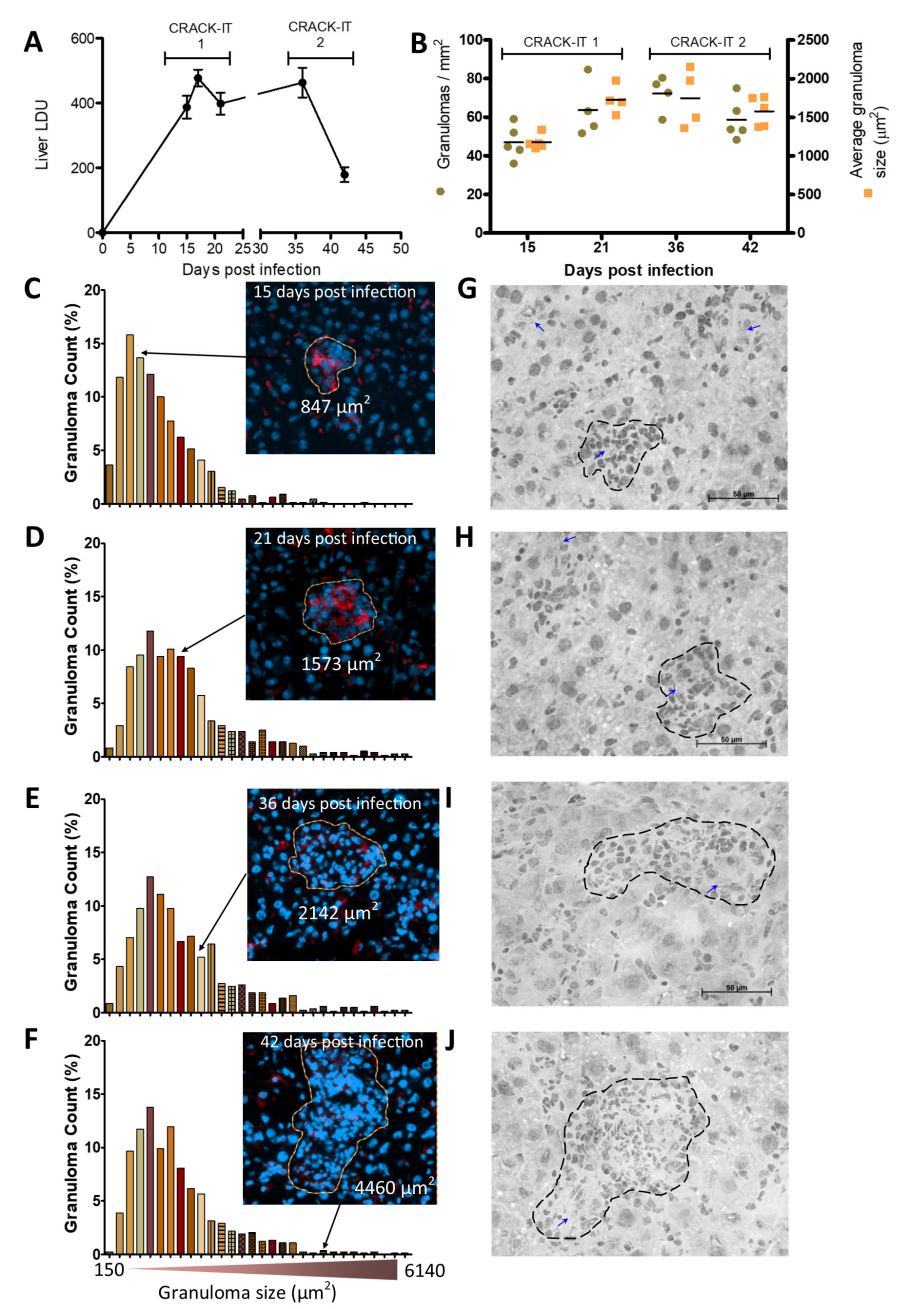
Hepatic response to *L. donovani* infection. Livers from BALB/c mice infected with *L. donovani* were removed at 15-, 17-, 21-, 36- and 42-days post infection. **A**. Parasite burdens were determined from impression smears and are represented as mean LDUs ± SEM. **B**. Granuloma density (mm ^2^; green dots) and average granuloma size (µm ^2^; yellow squares) are shown per mouse and with group mean. **C**– **F**. Distribution plot of granuloma size (ranging from 150 µm ^2 ^to 6140 µm ^2^) for 15, 21, 36 and 42 days p.i. respectively. Inset shows representative granulomas identified using TissueQuest, with F4/80 staining (red) and attributed size in µm ^2^. **G**– **J**. Granuloma morphology at 15, 21, 36 and 42 days p.i. respectively. H&E x40 magnification; scale bars 50 µm. Blue arrows indicate amastigote clusters. Dashed line denotes granuloma perimeter.

**Figure 7.**
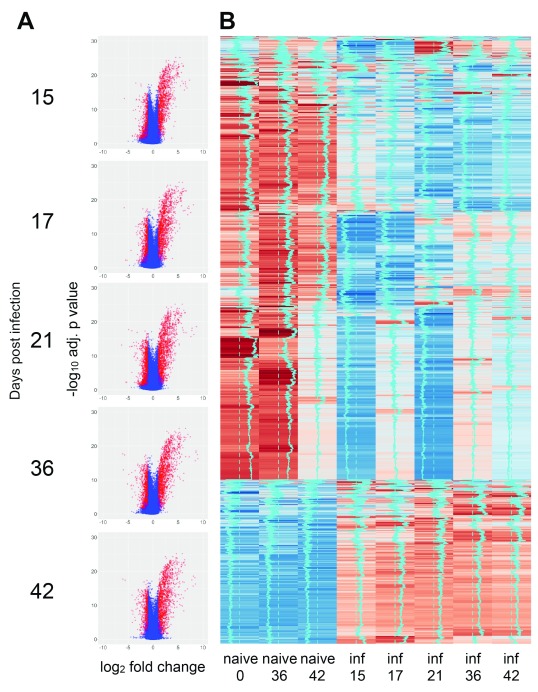
Transcriptomic profiling of hepatic response to *L. donovani* infection in BALB/c mice. **A**. Volcano plots showing DE probes at each time point of infection (relative to matched naïve control mice). Data are shown as Log2 FC in expression against Log10 adjusted p value. **B**. Heat map of probe expression intensity across time series. Data are pooled for n=5 mice per group for clarity. Vertical blue trace indicates mean intensity signal. Sample “naïve 0” was used to calculate DE genes for days 15, 17 and 21 p.i. Samples “naïve 36” and “naïve 42” were used to calculate DE genes for their respective time points p.i.

Finally, given its importance to the host response to infection, we examined the status of macrophage activation, and observed a good degree of concordance for M1-associated transcripts (as described in Figure 6 of 41) between hamster and mouse (55% of genes, including *Ccl2*, *Cxcl9*, *Cxcl10*, *Ifng*, *Irf1*, *Irf7*, *Irg1*, *Socs3*, *Stat1 and Ccr7*). In contrast, M2-associated genes were less commonly regulated in mouse compared to hamster spleen (23% of genes listed in Figure 7 of 41, including *Ccl2*, *Cd209a*, *Cebpb*, *Chi3l1*, and *Il1rn*). These data are in accord with the above analysis indicating an enhanced Th-2 type inflammation in hamster compared to murine EVL.

### Hepatic response to *L. donovani* infection in BALB/c mice

We adopted a similar approach to examine transcriptional changes in the liver of *L. donovani* infected mice, where the process of granulomatous inflammation has been well-characterised at the histological level ^13, 49, 50^. In contrast to the persistent parasite burden observed in the chronically infected spleen, parasite burden was significantly reduced in the liver by d42 p.i. ( Figure 6A). This was commensurate with the development and maturation of host protective granulomas, as indicated by quantitative morphometric analysis of granuloma number and area ( Figure 6B–F) and through observation of H&E stained sections ( Figure 6G–J).

In the infected liver, 16192 probes were scored as DE (>2 FC, 5% FDR) for at least one time point ( Figure 7 and Table S4). As seen in volcano plots ( Figure 7A vs Figure 2A) there was a tendency towards greater fold changes in mRNA accumulation compared to spleen with a bias towards up-regulated genes.

**Figure 8.**
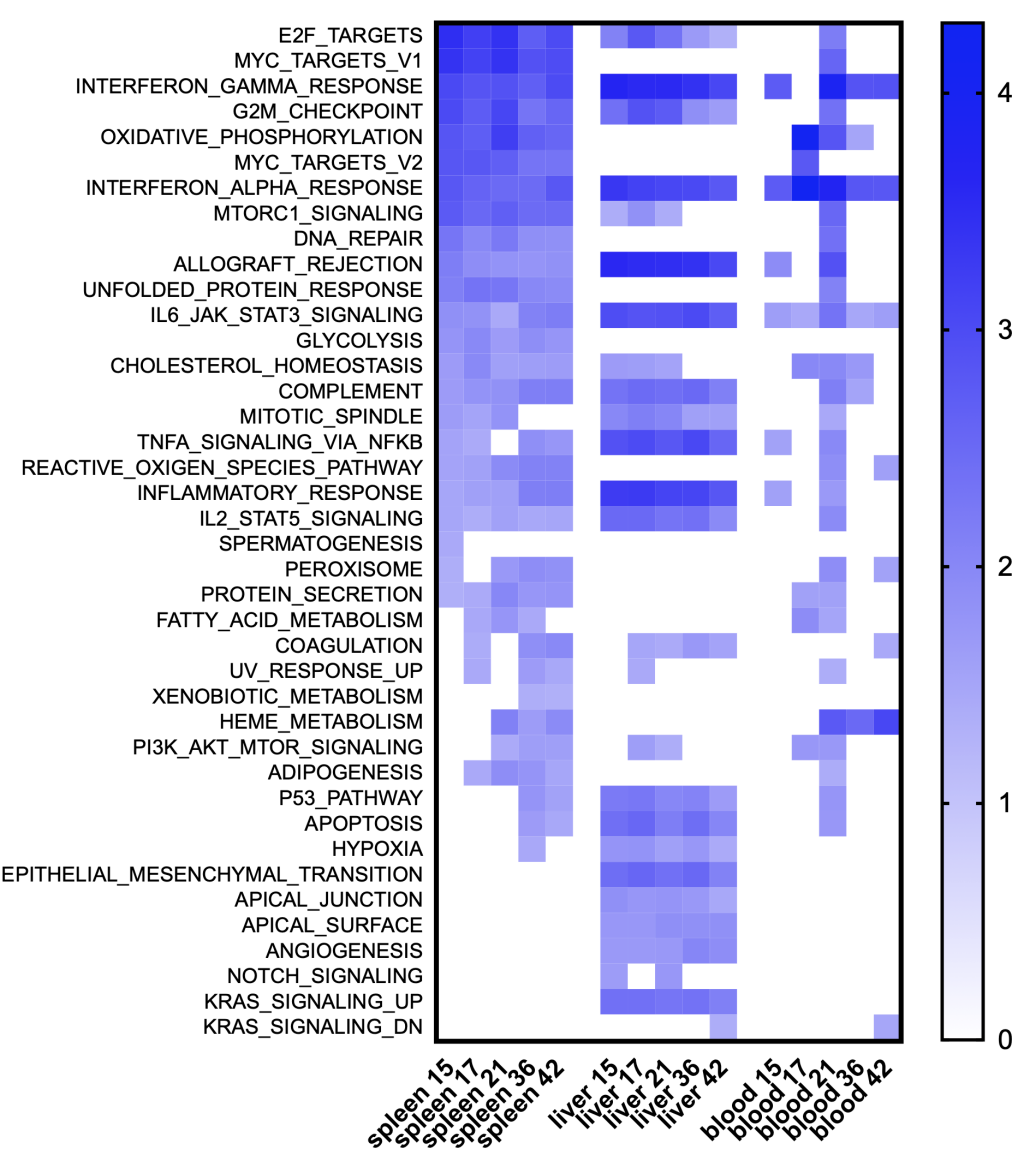
Comparison between hepatic and splenic responses to *L. donovani* by GSEA. Data were subjected to GSEA with reference to MSigDB Hallmark gene sets and normalized enrichment score (NES) over time is shown as a heat map for significant gene sets enriched in liver spleen and blood at the state time points.

By GSEA, we identified 20–24 hallmark gene sets that were associated with infection over the time course of infection (using a conservative threshold of 5% FDR); these enriched gene sets showed a high level of concordance and included allograft rejection, inflammatory response, IFNγ and IFNα signalling, TNF- , IL-2- and IL-6-related signalling pathways, pathways related to cell cycle, apoptosis, and complement Several were also found associated with the response to infection in the spleen ( Table 1 and Figure 8). There was, however, liver-specific enrichment for hallmark gene sets representing regulation of *Kras* signalling, epithelial and mesenchyme transition, angiogenesis, notch signalling and apical junction and apical surface. The latter are likely to reflect underlying changes in hepatocytes, which although not grossly abnormal in structure in H&E sections, may nevertheless have an important role in liver pathophysiology during disease ^51^.

**Figure 9.**
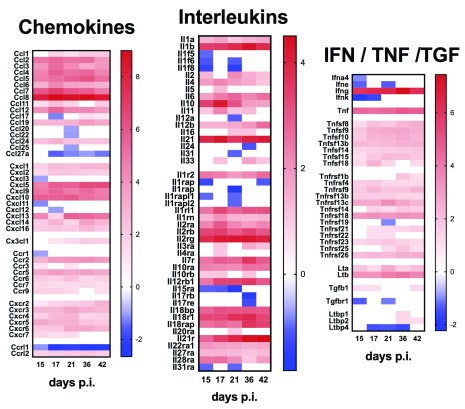
Cytokine and chemokine ligand and receptor gene expression in the liver of mice infected with *L. donovani*. **A**– **C**. Heat maps representing Log2 fold change in mRNA abundance for chemokines, interleukins and IFNγ / TNF family members and their receptors are shown. Only genes that were DE for at least one time point post infection are shown. Blue blocks represent down-regulated genes and red blocks indicate up-regulated genes. White blocks represent genes that were not significantly DE at a given time point.

mRNA accumulation for genes associated with cytokines, chemokines and their associated receptors were also compared over time ( Figure 9). In general, the breadth and intensity of response seen in the liver was greater than in the spleen, a result at least in part due to the greater change in cellular composition between resting and inflamed liver compared to resting and inflamed spleen. Nevertheless, it is also likely that some of the cytokine / cytokine receptor pathways that are more pronounced in the liver may reflect the self-cure phenotype associated with this organ. For example, at least at the level of mRNA abundance, IFNγ and IL-10 are not discriminatory between spleen and liver, whereas early increases in mRNA abundance for TNF and a broad range of TNF superfamily members appears liver specific. Of note, for many cytokines and chemokines, day 36 represented the peak of the response in the liver, with reduced mRNA accumulation at day 42 associated with the reduction in parasite load seen at this time ( Figure 6).

**Figure 10.**
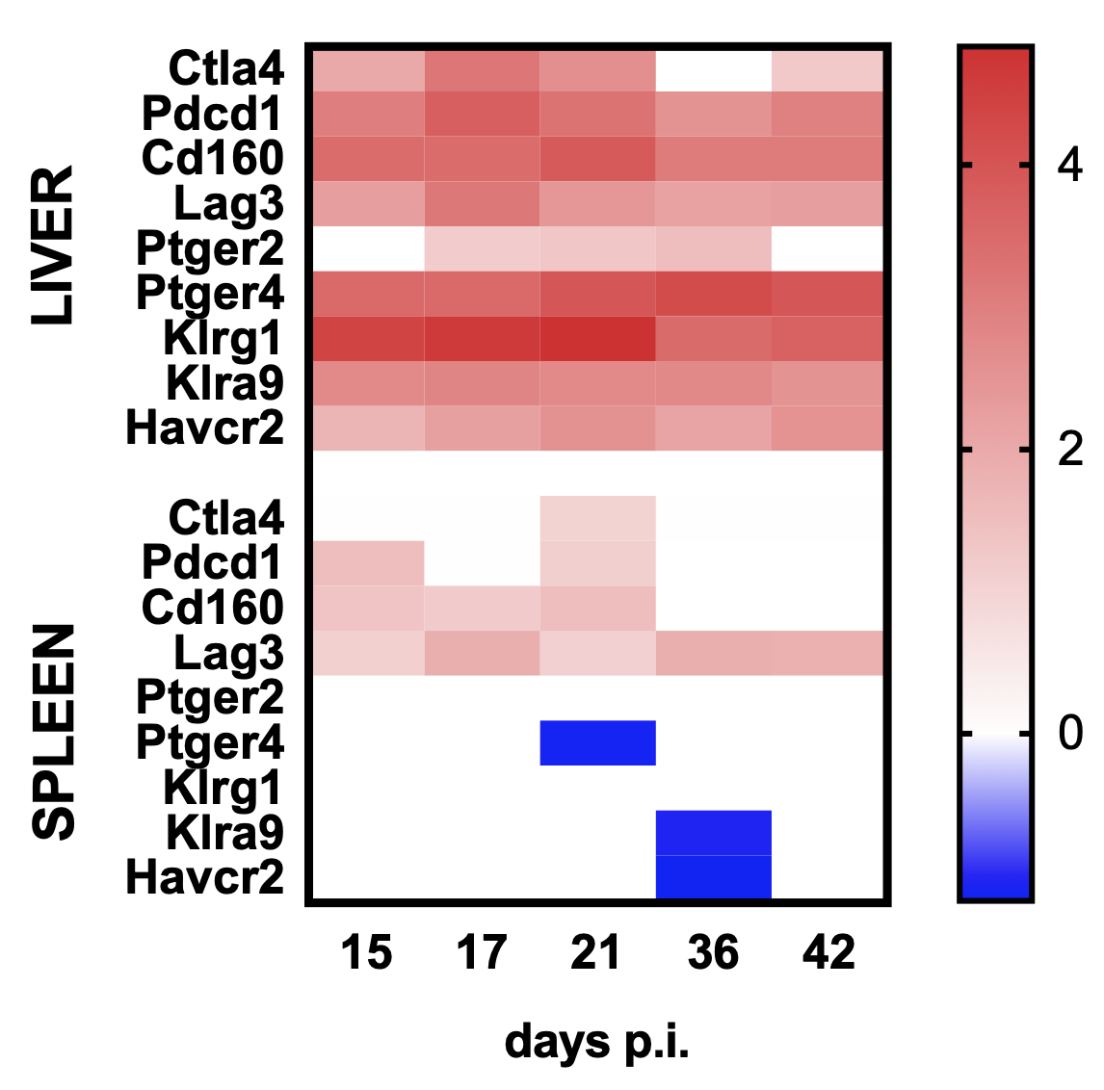
Expression of immune checkpoint genes in the spleen and liver of mice infected with *L. donovani.* Heat map representing Log2 fold change in mRNA abundance for immune checkpoint genes in spleen and liver of *L. donovani*-infected BALB/c mice. Only genes that were DE for at least one time point and at least one organ are shown. Blue blocks represent down-regulated genes and red blocks indicate up-regulated genes. White blocks represent genes that were not significantly DE at a given time point.

Finally, we examined the expression of inhibitory receptors associated with T cell exhaustion ^5, 52, 53^, to determine whether expression at the whole tissue level provided any clues as to the rate of cure for *L. donovani* infection seen in these two organs ( Figure 10). Inhibitory receptors were more prominently associated with the hepatic response, again likely reflecting the more dramatic increase in relative T cell frequency in liver vs. spleen. In spleen, *Lag3* was the only inhibitory receptor that remained elevated over the entire course of infection. Of note, similar to mouse spleen, *Lag3* was also significantly upregulated in hamster spleen whereas *Havcr2* (Tim-3) was also significantly down-regulated ( Table S3).

### Whole blood transcriptional response to *L. donovani* infection.

**Figure 11.**
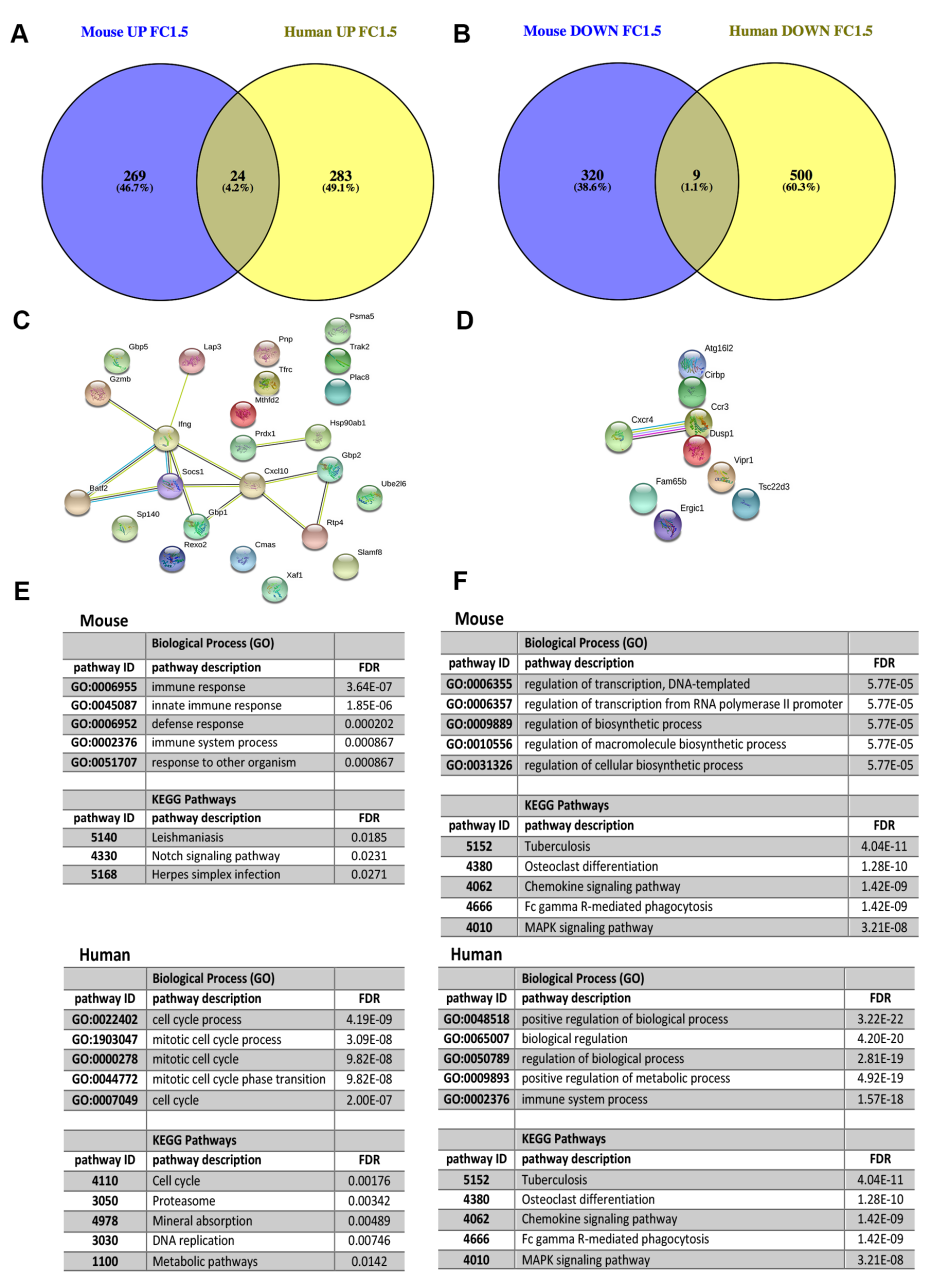
Comparison of whole blood transcriptome in murine EVL and human VL. **A** and **B**. Murine whole blood data from day 36 p.i. was re-analysed with a cut off of log2FC= 1.5 to make comparable with the human data presented in 39. Venn diagram indicates a minimal overlap between DE genes that are up regulated ( **A**) and down-regulated ( **B**) during infection. **C** and **D**. STRING analysis of the common up-regulated ( **C**) and down-regulated ( **D**) genes. **E** and **F**. Most significant (by FDR) GO terms and Kegg pathways associated with mouse and human specific up-regulated ( **E**) and down-regulated ( **F**) genes, generated by STRING.

As whole blood is the most accessible target tissue for transcriptomic profiling in humans and previous work had suggested that whole blood signatures might be useful avatars of the systemic response, we compared the response between spleen, liver and blood. 26,836 probes (12348 annotated genes) were identified as DE (FDR 0.05, FC2) in at least one time point ( Table S5). By GSEA blood was, not surprisingly, more similar to spleen than liver ( Figure 8). We next took advantage of a recently published whole blood transcriptomic analysis ^39^ to compare responses across murine EVL and human VL. Using day 36-infected mice as a comparator, our results suggest a high divergence in whole blood response between EVL and human VL ( Figure 11).

### Identification of common response signature in spleen, liver and blood during murine *L. donovani* infection

**Figure 12.**
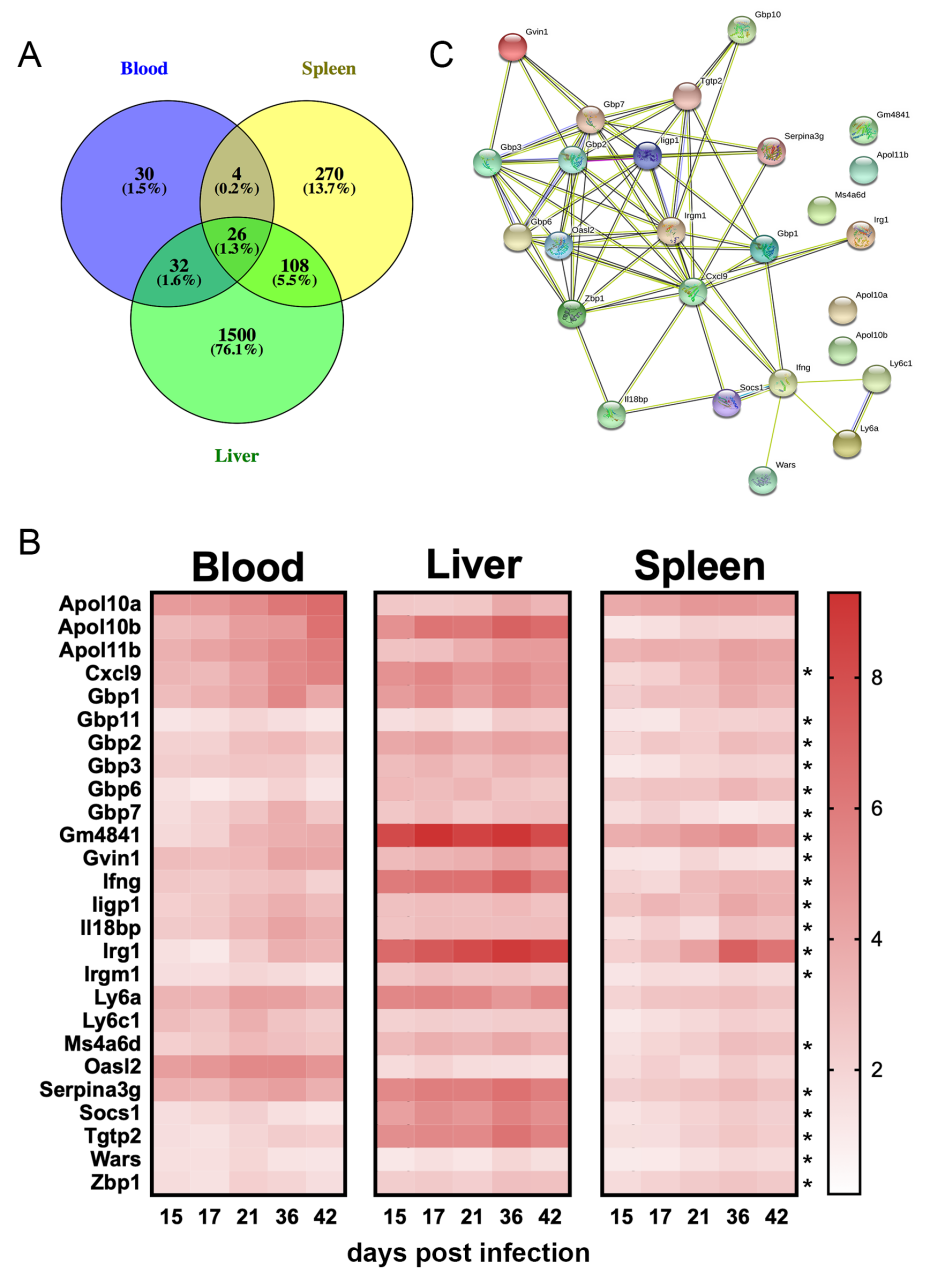
A 26-gene transcriptional signature is present in blood, spleen and liver throughout the course of *L. donovani* infection in BALB/c mice. **A**. Venn diagram showing relationship between genes consistently up-regulated across time in blood, spleen and liver. **B**. Heat map showing expression over time for the 26 gene signature in each tissue. Data are shown as Log2FC, indicated by the scale bar. Asterisks denote that genes were also upregulated in the spleen of hamsters infected for 28 days (data from 41.) **C**. STRING analysis of the 26-gene signature indicates two hub genes ( *Cxcl9* and *Gbp1*) forming a network that encompasses 21/26 (81%) of these genes.

Finally, we asked whether it was possible to identify a common response signature found in all tissues that might act as a biomarker of infection and which, when measured in blood might reflect or predict systemic disease. When analysed for genes that were DE at all time points in all tissues, we did not identify any genes that were significantly down regulated in expression. In contrast, 26 genes were consistently and significantly up-regulated across blood, spleen and liver at all times p.i in murine EVL ( Figure 12). These genes, of which 25/26 (96%) are interferon responsive, clustered in STRING analysis around 2 nodes ( *Cxcl9* and *Gbp1*: Figure 12B and C) and reflected a significant enrichment for GO TERMS including “cellular response to interferon-beta” (GO:0035458, FDR 7.19×10 ^-8^), “defence response to protozoans” (GO:0042832, FDR 7.22×10 ^-8^), “immune response” (GO:0006955, FDR 9.71×10 ^-8^), and symbiont containing vacuole” (GO:0020003, FDR 2.15×10 ^-9^). The gene set also contained 3 apolipoprotein L genes ( *Apol10a*, *Apol10b* and *Apol11b*) which are phylogenetically related amongst the 12 members of the murine Apolipoprotein L family ^54^. Of note, 19/26 (73%) genes in this signature were also upregulated in hamster spleen ( Figure 12B), whereas 6/26 (23%) were upregulated in human whole blood ^39^. Common to all data sets were genes encoding IFNγ, the CD20-like antigen membrane spanning 4-domains A6A (MS4A6A/ *Ms4a6d*), and the interferon-inducible genes guanylate binding protein 2 (GBP2/ *Gbp2*), suppressor of cytokine signalling (SOCS1/ *Socs1*) and tryptophanyl tRNA synthetase (WARS/ *Wars*).

## Discussion

The immune response to *L. donovani* infection has been extensively analysed using a wide array of technologies, including use of gene KO mice, advanced imaging techniques and cell/cytokine manipulations ^5, 8, 50, 55– 57^. Yet a comprehensive picture of the dynamic complexity of the host response has not previously been reported. Here, we provide data from a detailed transcriptional analysis of the response of BALB/c mice to *L. donovani* infection and compare this where possible to existing data sets from other species, including humans. This high-level overview of transcriptional changes associated with infection poses a number of important questions and provides an important additional data resource for the leishmaniasis research community.

BALB/c mice were chosen for this study due to their extensive use as an immunological model of infection and as a tool for the pre-clinical evaluation of drugs and vaccines ^58– 61^. In this and most other mouse strains on a *Slc11a1* (Nramp1) mutant background, such as C57BL/6, hepatic infection is self-resolving and associated with the development of granulomatous inflammation, whereas splenic parasite load persists, often for several months, accompanied by marked alterations to organ microarchitecture ^13^. We selected time points when hepatic and splenic parasite burden were increasing and also to reflect the time frame over which a dichotomy in host resistance becomes apparent in spleen and liver. Where possible, we have also mined existing data to look for similarities and/or differences in transcriptional response. Whilst these comparisons are informative, some degree of caution needs to be exerted on their interpretation. Platforms used for analysis of transcriptomic changes differ (Agilent gene arrays, Illumina Bead Chip arrays, RNA-Seq), with microarrays often having more limited dynamic range than RNA-Seq. For this reason, we have not attempted any quantitative comparison in expression levels across platforms. Rather, our comparisons are drawn on the basis of comparing DE gene lists generated using standard analysis and statistical tools suited for each platform. In addition, variables such as tissue parasite load and time of infection are difficult to standardise (particularly for human disease where the time of infection is usually unknown), and with the exception of our study, data is currently restricted in most species to a single tissue site. It should also be remembered that whether comparing between tissues in the same host or across species, lists of DE genes may reflect changes due to either alterations in gene expression pattern of individual cells or due to changes in tissue cell composition. Although in some cases our results were unexpected and showed dissimilarity between experimental models and human disease, this should not be taken as an argument against the value of research in such models. Rather, it stresses that once proof-of-concept has been achieved in a model, additional validation based on human data is essential. Furthermore, such human data may form the basis for selecting an appropriate pre-clinical model for the question at hand, or indeed for engineering more refined models of novel aspects of human disease.

Splenic histopathology of infected BALB/c mice, as expected, indicated extensive remodelling of the splenic architecture and the late onset of extramedullary haematopoiesis, similar to that seen in other mouse strains ^8^. Coincident with these changes, we observed transcriptional signatures associated with the activation of immune responses, angiogenesis and coagulation pathways. Interferon-related pathways were highly dominant. Approximately 13% of the identified DE genes were predicted to be downstream of IFNγ signalling and ~30% of all genes were scored as interferon inducible using the Interferome database ^46^. IPA identified two candidate negative regulators IL-10Ra and TRIM24. IL-10 signalling through IL-10Ra (CD210) is well recognised for its role in inhibiting immune clearance of *Leishmania* in mouse models and in humans ^1, 2, 7, 62– 64^, operating to directly regulate macrophage activation and also the function and development of IL-10 producing Th1 and Tr1 cells ^1, 63– 66^. Therapeutic interventions to target IL-10 mediated immune regulation in human VL have been posited ^67^. TRIM24 is a bromodomain-containing transcriptional regulator protein that has recently been the focus of attention as a regulator of STAT3 recruitment during oncogenesis ^68^ and as a positive regulator of Th2 cytokine production in Th2 cells ^69^. The predicted inhibition of TRIM24 would, therefore, be consistent with a dominant Th1 type immune response during murine EVL. TRIM24 is also observed as a target for inactivation during infection with lab-adapted H1N1influenza A virus ^70^ and has been shown to be targetable using novel heterobifunctional protein degraders ^71^, suggesting that pathways controlled by TRIM24 could be further manipulated in favour of host protection.

In contrast to the murine EVL model, data generated by Melby and colleagues suggests that the immune response in hamster EVL favours a more prominent Th2 like response ^40, 41^. In addition, their analysis also indicates that macrophages in hamster EVL are conditioned to respond to activation with the induction of a program of gene expression that favours amastigote growth, including *Arg1*, *Ido-1* and *Irg1* and associated with STAT3 activation ^41^. At the peak of splenic infection in our study (d36 p.i.), which we considered the best approximation of the hamster model that we could achieve based on the degree of pathology, we did not observe increased accumulation of *Ido1* in murine EVL and a comparison of signature M1 and M2 genes suggested that in murine EVL, M1 activation was favoured. At a tissue level, alternate regulators of inflammation such as *Ptsg2* and *Il-10* appeared to be favoured in murine EVL. Upregulation of *Ptsg2* (Cox2) is consistent with the enhanced production of prostaglandins in spleen cells from mice with *L. donovani* infection ^72^ and with the host protective effect of Cox2 inhibition *in vivo* ^73^.

There has been considerable recent interest in the potential for host-directed therapies in different forms of leishmaniasis ^4, 5, 67, 74– 77^. Of interest, therefore, is how well pre-clinical models may predict novel approaches applicable to human VL. Our analysis showing increased mRNA abundance for a variety of checkpoint inhibitors including CTLA4, PD1, LAG3 and others ( Figure 10) supports the notion that these may be candidates for the development of immunotherapy, along with key suppressive cytokines like IL-10. In the mouse, these checkpoint inhibitors (along with many cytokines, chemokines and their receptors) have a complex pattern of temporal and tissue-specific regulation which, together with the potential for cell migration between sites, makes predictions concerning the impact of immunotherapy challenging. Some of these checkpoint targets are equally represented in mouse and hamster spleen and human whole blood e.g. LAG3 and CTLA4, whereas others show species restricted regulation. For example, IDO-1 is up regulated in hamster spleen and human whole blood, but not in any mouse tissue examined. Importantly, these diverse data as well as the clear dichotomy of expression at a tissue specific level, indicate the need to build a more comprehensive and uniform knowledge base regarding these pathways in different models and forms of human disease.

Analysis of whole blood has been a valuable tool for monitoring transcriptional changes associated with infection and even for predicting drug efficacy ^19^, but the relationship between transcriptomic changes in blood and tissue remains poorly understood. Pooling data across all tissues and time points, we were able to identify a 26-gene signature that was common to spleen, liver and blood during murine VL. 96% of these genes have been reported as interferon inducible, clearly indicating the dominant nature of interferon signalling during murine VL and that this common signature reflects disease in all organs/tissues studied. The value of defining signatures of this type might be to understand common pathways underlying inflammation or to provide new biomarkers that reflect the level of ongoing systemic disease that can be monitored in blood. We will further elaborate on the use of this signature to evaluate chemotherapy induced cure in murine VL in a subsequent manuscript (Forrester *et al.* manuscript in preparation). The clinical value of this signature is however questionable at this stage, given the lack of similarity between whole blood transcriptomic responses observed in our study and that of humans with VL in Brazil ^39^. In a similar study in acute melioidosis, 26.9% of genes were similarly regulated in murine and human infection ^21^. The reasons for this lack of similarity in VL are likely to be multiple, including the chronicity of infection, difference in pathogen ( *L. donovani* vs. *L. infantum*), specific characteristics of Brazilian VL compared to VL on other continents ^12^ or differing analytical methods. It would be informative, given the above discussion, to compare human whole blood from a greater range of patient populations with VL to better understand the extent of temporal and geographic diversity in the transcriptome of patients with VL. Likewise, data on hamster whole blood might help ascertain whether the blood transcriptome in this model more closely resembles that found during human disease.

Finally, a deeper understanding of subtleties of the immunopathology of leishmaniasis in different target tissues (e.g. granulomatous vs. non-granulomatous) and their relationship to gene transcriptional changes will require more in-depth analysis than that reported here, using new techniques to integrate image analysis with complex “omics” and other meta data. Such approaches are fuelling significant advances in precision medicine in other fields, notably cancer and neuroscience ^78– 80^, but have to date had limited application in the field of infectious disease. To facilitate the development of such approaches in leishmaniasis, we have generated a digital whole slide collection of the tissue sections generated in this study, stained with both H&E and markers to identify myeloid cells (F4/80 and 1A8). These are available via a new network designed to support the development of digital pathology in leishmaniasis ( www.leishpathnet.org), and improve transparency and data sharing. In addition, the data collected from the animals used in this study forms part of a larger program of work (the CRACK IT Virtual Infectious Diseases Research Challenge; https://crackit.org.uk/challenge-16-virtual-infectious-disease-research) that had the goal of generating an *in silico* model of the immune response during murine EVL, with the aim of reducing the number of animals required for basic research on immune regulation and for evaluating potentially novel therapeutic interventions ( www.leishsim.org). Future manuscripts from the CRACK IT program will extend the transcriptomic analysis presented here to include comparative studies with *L. infantum* infection, the response of *L. donovani*-infected BALB/c mice to chemotherapy, and the response to *L. donovani* infection in C57BL/6 (B6) and B6. *Rag1* ^-/-^ mice.

## Data availability

Microarray data is available via the Gene Expression Omnibus (Ascension number GSE113376: https://www.ncbi.nlm.nih.gov/geo/query/acc.cgi?acc=GSE113376).

**OSF: CRACKIT Virtual Infectious Diseases project.** Supplementary Table 1– Supplementary Table 5 (see information below in Supplementary material) and raw data for Figure 1A and B and Figure 6A–F are available, https://doi.org/10.17605/OSF.IO/9WSDK ^81^.

Data on OSF are available under the terms of the Creative Commons Zero "No rights reserved" data waiver (CC0 1.0 Public domain dedication).

Whole slide images and individual mouse metadata are available from www.leishpathnet.org (study designations CRACKIT-1 and CRACKIT-2). Requests for access to tissue samples from these studies will be accommodated where possible and subject to availability.

RNA-Seq data from a hamster model of VL is available from: https://www.ncbi.nlm.nih.gov/geo/query/acc.cgi?acc=GSE91187

Blood transcription of human infection are available from: https://www.ncbi.nlm.nih.gov/geo/query/acc.cgi?acc=GSE77528

## Supplementary material

**Supplementary Tables 1–5** are available here: https://doi.org/10.17605/OSF.IO/9WSDK ^81^

**Table S1. 9634 DE probes across time course of *L. donovani* infection in the spleen of BALB/c mice.** Sheet 1 provides the list of 9634 DE probes identified on the basis of FDR of 0.05 and a FC of 2 for at least one infection time point vs naïve control. Sheet 2 provides the collapsed 5096 DE gene list. Expression data are shown as Log2FC.

**Table S2. GSEA analysis of DE genes in the spleen of *L. donovani* infected BALB/c mice.** Data in the Summary tab was used to generate Table 1 and Figure 3A.

**Table S3. Lists of annotated DE genes in mouse and hamster spleen following *L. donovani* infection.** Gene lists are arranged as DE in both hamster and mouse, hamster DE only and mouse DE only. Expression data are shown as log2FC.

**Table S4. 16192 DE probes across time course of *L. donovani* infection in the liver of BALB/c mice**. Sheet 1 provides the list of 16192 DE probes identified on the basis of FDR of 0.05 and a FC of 2 for at least one infection time point vs naïve control. Sheet 2 provides the collapsed 8033 DE gene list. Expression data are shown as Log2FC.

**Table S5. 28636 DE probes across time course of *L. donovani* infection in the blood of BALB/c mice**. Sheet 1 provides the list of 28636 DE probes identified on the basis of FDR of 0.05 and a FC of 2 for at least one infection time point vs naïve control. Sheet 2 provides the collapsed 12348 DE gene list. Expression data are shown as Log2FC. Sheet 3 contains DE genes identified by Gardinassi *et al.* from whole blood of VL patients ^39^.
