# Supplementary material for: Tissue and host species-specific transcriptional changes in models of experimental visceral leishmaniasis
Source: Wellcome Open Res. 2019 Jan 2;3:135. Originally published 2018 Oct 29. [Version 2] doi: 10.12688/wellcomeopenres.14867.2 (PMC6248268; doi:10.12688/wellcomeopenres.14867.2)
Supplement: Peer review history

# Peer review: Referee response for version 2

**DOI:** 10.21956/wellcomeopenres.16356.r34507

**Published:** 2019-01-09

**Authors:** Washington Luis Conrado dos-Santos, Caroline Vilas Boas de Melo

**Affiliations:** 1 Fundação Oswaldo Cruz, Instituto Gonçalo Moniz, Salvador, Brazil; 2 Fundação Oswaldo Cruz, Instituto Gonçalo Moniz, Salavador, Brazil

The authors satisfactorily dealt with all the questions placed in our previous analysis.

 We have no further comments to make.

We have read this submission. We believe that we have an appropriate level of expertise to confirm that it is of an acceptable scientific standard.

# Peer review: Referee response for version 1

**DOI:** 10.21956/wellcomeopenres.16203.r34134

**Published:** 2018-11-22

**Authors:** Philip Scott

**Affiliations:** 1 Department of Pathobiology, School of Veterinary Medicine, University of Pennsylvania, Philadelphia, PA, USA

This study provides an analysis of transcriptional changes occurring in the *Leishmania donovani* infected mouse over time in the spleen and liver. There is no comparable kinetic analysis of transcriptional changes in visceral leishmaniasis, thus indicating the significance of the work. The authors also attempt to compare their findings with published transcriptional analyses in hamster and human VL, which is useful. Overall, the study is well-done with some important caveats that the authors are aware of and which they mention in the text. First and foremost, the kinetic study in mice is a combination of two experiments and the authors might be clearer in how they handled the potential batch effects that one might anticipate seeing. Looking at Figure 2 it would appear that there are significant differences between the first experiment (Day 15, 17 and 21) and the second experiment (day 36 and 42).  Second, a comparison between data generated by microarray and RNAseq will have several notable issues, and while the authors mention this issue they might expand a bit on how those differences will influence interpretation of their results. It can be useful to make these comparisons, but critical to ensure the readers understand the significant caveats. Finally, it would be helpful to have a clearer discussion of what they have found out about tissue differences in response to infection between the spleen and liver, and how those differences might be responsible for differences in the parasite control that is seen in these two tissues.

 The minor problem that becomes a bigger problem as it is evident in many of the figures is the inability to read several of the figures because of font size, as well as some figures that may not be informative for the readers. For example: Fig. 1C-D: arrows almost invisible: Fig. 3A. difficult to evaluate; Fig. 3C- Font size; Fig. 3E- Font size; Fig 5B (a really cool figure)- but font size too small for the labeled genes; Fig. 5C-F- difficult to interpret; Fig. 11C, D- Font size; Fig. 12C- Font size. One additional question: There appear to be no changes in the granulomas in Fig. 6B from day 21 to 42. Is this expected?

 In summary, in spite of the caveats mentioned above, this study provides data that can be mined by others to better understand the immune responses in VL. 

I have read this submission. I believe that I have an appropriate level of expertise to confirm that it is of an acceptable scientific standard.

# Response

**Published:** 2018-12-17

**Authors:** Paul Kaye

**Affiliations:** Department of Biology and Hull York Medical School, Centre for Immunology and Infection, University of York, UK

*1. batch effects resulting from conducting two independent experiments*

 We are aware of the possibility of batch effects and have tried to control for these as best as possible. The size and scale of the experiments performed for this study (and also to support additional analysis to be described elsewhere) made it logistically impossible to use a single cohort processed on the same day.  We confirmed health status across cohorts according to FELASA guidelines and had matched “naïve” control mice for each cohort (and for each time point in CRACKIT_2). Differential gene expression was conducted relative to these matched controls rather than to a common “naïve” group to further reduce batch effects. These details are described in the M&M and Results.  

*2. issues resulting from comparison of RNA-Seq to microarray*

 We have expanded our discussion on this point as suggested by the referee

*3 tissue specific differences in parasite control in spleen and liver*

 We have included some additional comments on this issue throughout the manuscript.  However, as this data represented the platform on which to base further mechanistic studies, we have refrained from making too many conclusions purely on expression data.

*4. font size of figures*

 We have reviewed all the figures for legibility and where necessary either increased the font size, of re-designed the figure to improve readability. For the STRING diagrams in Figs 11 and 12, the main aim is to show the extent to which different groups of DE genes are clustered. We tried increasing font size, but this actually makes the figure less clear due to proximity of each name. We think it valuable for readers to be able to view the gene names (even if requiring zoom on a pdf version of the manuscript and so have retained a small font on Figs 11 and 12).

*5. was no difference in granulomas between d21 and d42 expected?*

 There are some subtle differences in the size distribution over time, and at d42 there is already some killing of parasites within granulomas (as evident from Fig 1A). This is not fully reflected in change in granuloma size, however.

# Peer review: Referee response for version 1

**DOI:** 10.21956/wellcomeopenres.16203.r34135

**Published:** 2018-11-20

**Authors:** Syamal Roy

**Affiliations:** 1 Cooch Behar Panchanan Barma University, Cooch Behar, West Bengal, India

The manuscript entitled “Tissue and host species-specific transcriptional changes in models of experimental visceral leishmaniasis” by Ashwin *et al.* describes tissue specific alteration in transcriptome in *Leishmania donovani* infected Balb/c mice in a longitudinal infection and compares the results with those from other species.

 The number of DE probes was studied between normal and infected splenic tissues of Balb/c mice as a function of time, peaking at d36.  After removal of multiple probes, the number of annotated genes was 5096 set across all time points. The most commonly enriched genes were IFN-Y, TNF-α signalling, IL-6-JAK/STAT signaling, E2F targets, KRAS signaling and others. Interestingly these changes are coupled with extra medullary myelopoiesis in spleen. IPA showed significant upstream regulators predicted to be up regulated during infection whereas IL-10RA and TRIM24 were upstream regulators that were predicted to be down regulated, despite not being DE themselves. Using the Interferome database they could show that 32.3% of all DE genes scored as interferon inducible. Further pathway analysis was done to find upstream regulators of the DE genes. The mouse data was compared with the hamster and human data available in the literature. It is also highly appreciable that the data collected from the animals used in this study forms part of a CRACKIT Virtual Infectious Diseases Research Challenge program that focuses on generating an in silico model of the immune response during murine EVL in order to reduce the number of animals required for basic research on immune regulation and for evaluating potentially novel therapeutic interventions. Besides this, they have also made Microarray data accessible in the public domain through Gene Expression Omnibus a part of NCBI Databases.

 And finally, they have confirmed that there is marked tissue-specific alteration in the transcriptome of infected mice over time and also identified commonality and differences between murine, hamster and human responses to infection that were previously unrecognized. They have shown the commonality of interferon-regulated genes while confirming a greater activation of type 2 immune pathways in infected hamsters compared to mice, and also pointed out that Cytokine genes and genes encoding immune checkpoints were markedly tissue-specific and dynamic in their expression and pathways. Through data, they have made an effort to address the value of measuring peripheral blood transcriptomics as a potential window into the underlying systemic disease. They could show a 26-gene signature that is common to spleen, liver and blood during murine VL. Oddly enough, there is a lack of similarity in whole blood transcriptomic response between mouse and human systems.

 The major challenge now is the absence of an experimental model that would mimic the spectrum of human disease. Studies on the animal model provide us with a wealth of information about the host-parasite interaction, but there is no such model available that would reproduce human disease. Thus, so many questions remain unanswered in order to design control strategies. This observation re-enforces the notion that there is a gap between experimental data and human disease on leishmaniasis.

 General comments:

1. The authors try to compare DE transcripts at d28 post infection from naïve hamsters to L. donovani to 36 days post infected mice and their study is based on the best match (page 9 of 21). It will be nice if the basis for the match criterion can be elaborated.
2. Figure 5A is a Venn diagram showing the overlap of DE genes in page 10. It looks like a weighted Venn diagram and the authors have cited Venny 2.1 for making Venn diagram but I wonder if the weighted Venn cannot be formed by Venny 2.1.  It will be helpful if the tool or package that has been utilized for its formation can be included. In case it is just a normal Venn diagram then the figure requires an edit.
3. Figure 5B: Please provide the citation to the tool or package that has been utilized for the correlation plot of Log2FC for mouse and hamster DE genes.
4. There are a few issues like parasite stain, duration of infection and resulting host-pathogen interaction etc that are beyond the authors’ control. This raises the question whether even monkeys can be used for testing vaccine candidates as little is known if the immune response to leishmania is similar to that observed in human.

Nevertheless as a whole I think it is a great effort by the group. It is a pleasure to read the article.

I have read this submission. I believe that I have an appropriate level of expertise to confirm that it is of an acceptable scientific standard.

# Response

**Published:** 2018-12-17

**Authors:** Paul Kaye

**Affiliations:** Department of Biology and Hull York Medical School, Centre for Immunology and Infection, University of York, UK

*1. elaborate on why mouse d36 and hamster d28 was considered “best match” for analysis*

 We have amended the text in the Results to indicate “at the peak of splenic infection” rather than “best matched” and made a further comment to this effect in the Discussion. 

*2. software to generate weighted Venn diagram in Figure 5A*

 We have added this information to the M&M

*3. software to generate correlation plot in Figure 5B*

 We have added this information to the M&M

# Peer review: Referee response for version 1

**DOI:** 10.21956/wellcomeopenres.16203.r34131

**Published:** 2018-11-15

**Authors:** Washington Luis Conrado dos-Santos, Caroline Vilas Boas de Melo

**Affiliations:** 1 Fundação Oswaldo Cruz, Instituto Gonçalo Moniz, Salvador, Brazil; 2 Fundação Oswaldo Cruz, Instituto Gonçalo Moniz, Salavador, Brazil

This is a very informative paper that compares gene expression during the early course of EVL in mice and hamsters, the differential expression pattern in spleen, liver and whole blood and the experimental data with the available data obtained from HVL. The strategy used is robust and very transparent. The results are in accordance with the methodology used. It will help to define new strategies for using of experimental models and to define new markers of immune response for HVL.

 METHODS:

 Why did the authors perform the study using two different experiments CRACK-IT_1 and CRACK-IT_2?

 Tissue transcriptomics: what impact may the early amplification and removal of outliers by normalisation have had on the comparative study presented in the manuscript?

 Was any additional normalization needed for comparisons performed between the data presented in this manuscript and those presented in references 39 and 41?

 RESULTS:

 Figures 1A and 1B_Raw (Supplementary data) The mean spleen weight of control animals at 36 days was ~4x higher than of animals with 14 and 42 days? Was the histology of these enlarged spleens similar to that observed in the other control animals? Do the authors think that including animals with enlarged spleen may have interfered with the data on gene expression?

 Figure 1C-1H:

 Technical details: For some reason the figures present some degree of opacity impairing adequate visualization. The blue arrows are so small that is difficult to find and the details such as infected macrophages are barely seen.

 For the usual reader the loss of red pulp/white pulp differentiation mentioned by the authors in the text may not be easily seen. A more representative image and addition of markers and legends indicating these morphological changes may help to present the data.

 Still about the figure comments presented in the text. Extramedullary splenic hematopoiesis is common in normal mice and has a tendency of increasing with age. Did the authors make any comparative quantitative assessment of spleen hematopoiesis between control and infected animals?

 Figure 6B: please replace “green square” with “green dots”.

 Figure 6E and 6F: I had the impression that there was a substantial decrease in F4/80+ cells in the granuloma. Was there a change in cell population in the granuloma at these stages of infection?

 Figure 10: It is interesting that Ptger4, Klrag9 and Havcr2 are upregulated in liver and down regulated in spleen. Can the author comment on this data?

We have read this submission. We believe that we have an appropriate level of expertise to confirm that it is of an acceptable scientific standard, however we have significant reservations, as outlined above.

# Response

**Published:** 2018-12-17

**Authors:** Paul Kaye

**Affiliations:** Department of Biology and Hull York Medical School, Centre for Immunology and Infection, University of York, UK

Methods

*1.  why two different experiments for the time course analysis?*

 The size and scale of the experiments performed for this study (and also to support additional analysis to be described elsewhere) made it logistically impossible to use a single cohort infected and processed on the same day.  To minimise batch effects, we confirmed health standard across both cohorts according to FELASA guidelines and had matched “naïve” control mice for each cohort (and for each time point in CRACKIT_2).  Differential gene expression was conducted relative to these matched controls rather than to a common “naïve” group to further reduce batch effects. These details are described in the M&M and Results.  

*2. impact of early amplification and removal of outliers by normalisation on the comparative study*

 There are multiple reasons to be cautious in conducting comparative studies with data derived from different platforms and we discuss these in the text.  It is difficult to know the significance of these effects, but we have erred on the cautious side e.g. avoiding direct quantitative comparisons and commenting only on DE genes identified by conventional platform specific approaches.

 3. was any additional normalisation used to compare mouse, hamster and human data sets?

 We have added a further statement to the M&M to clarify the text already in the Results section.   

 Results

*1. Fig1A/B Raw data on spleen size in naïve controls at d36*

 We thank the reviewer for pointing out this transcribing error in the raw data file. This has now been corrected (naïve spleen weight at d36 = 86+/-5 mg) 

 2. improve clarity of Figure 1C-H (arrows etc, demarcation of red and white pulp)

 The figures have been corrected as suggested to improve clarity

*3. comparison of spleen hematopoiesis between control and infected mice*

 We have not formally compared this in the current study, but a manuscript from our group related to this issue has recently been published (Preham et al CD4 ^+^T cells alter the stromal microenvironment and repress medullary erythropoiesis in murine visceral leishmaniasis. Front. Immunol. 2018. Doi: 10.3389/fimmu.2018.02958). 

*4. Figure 6B: replace green square with green dots*

 This has been corrected

*5. Figure 6E-F changes in granuloma cell populations over time*

 The F4/80 + DAPI images shown in Fig 1C-F were chosen to show representative granulomas of differing size and not to formally describe changes in cell populations over time. This type of analysis has been conducted previously e.g. Moore et al PlosOne 2012. 7(3):e34134 for B and T cells and Beattie et al PLoS Path 2010. 6(3):e1000805.   A further manuscript currently in preparation from the CRACKIT project (Hamp et al) will also contain further information on hepatic cellularity over time.  We have added an additional sentence to the discussion to highlight that transcriptomic changes as assessed here may be due to changes in cell composition or altered transcription levels within a given cell population. 

*6. Figure 10: comment on differential regulation of checkpoints in spleen and liver*

 We have included some additional discussion on these data and the difficulties in extrapolating to the effects of therapeutic blockade.

# Peer review: Referee response for version 1

**DOI:** 10.21956/wellcomeopenres.16203.r34133

**Published:** 2018-11-14

**Authors:** Yasuyuki Goto

**Affiliations:** 1 Laboratory of Molecular Immunology, Graduate School of Agricultural and Life Sciences, The University of Tokyo, Tokyo, Japan

Tissue-specific immune responses are always of interest in visceral leishmaniasis. The authors address this question by looking at transcriptomes of the spleen, liver and blood of infected mice and comparing them with infected hamsters and human VL patients. I feel that their approach is reasonable and the data shown here are very useful to the research field. It will be great if the authors can strengthen the conclusion by discussing/interpreting the data more in depth.

 Major comments

- The authors described in the Discussion that BALB/c were used in this study ‘due to their extensive use as an immunological model of infection…’. However, the authors’ group also use C57BL/6 mice for immunological studies of experimental VL. Is there any stronger reason to select BALB/c over C57BL/6?In fact, pathogenesis/immune responses during *L. donovani* infection may be different between those two inbred mice. For example, in the current study using BALB/c mice, the splenic parasite burden and spleen weight were almost parallel. It means the parasite number per host nuclei are relative stable over the course of infection. In contrast, C57BL/6 mice show higher increase in LDU over spleen weight, according to Ato M et al., Nature Immunology, 2002. Also, splenomegaly by *L. donovani* infection seems worse in BALB/c mice (~8 times enlargement at D35) than C57BL/6 mice (only 1.7 time at D28). These differences should be welcomed because they mean that comparison of the inbred mouse strains is also a viable approach besides comparison with other species.
- Although the title says tissue and host species-‘specific’ changes, I feel that the article more focuses on commonality among tissues and species. For example, the last part of this work focuses on the 26 genes commonly up-regulated in the spleen, liver and blood; what is the significance of identifying such genes? The authors know that at the time point kinetics of parasite proliferation in the spleen and liver are different, so what kind of phenomena should be associated with the commonly up-regulated genes? I believe that more careful watch into differently regulated genes, across tissues and across species, is indispensable. Identification of IDO-1 as a mouse-low, hamster-high and human-high factor is really intriguing and the authors are encouraged to take further analyses to identify such ‘protection-related factor candidates’ because it is supposed to be their objective.
- Related to the previous comment, representation of each tissue/species is a little vague. My understanding is; mouse spleen, vulnerable at D42; mouse liver, protective at D42; hamster spleen, vulnerable at D42; hamster liver, vulnerable at D42; human blood, reflecting systemic vulnerable condition. Is it correct and if yes what does mouse blood represent? Although I understand the authors know that it is not that simple, data analyses and discussions with clear and consistent hypotheses/assumptions are helpful to the readers.
- Page 4: Discussions on IL-10Ra and Trim24 are difficult to understand. ‘Upstream regulators that were predicted to be down regulated, despite not being DE themselves’, do you mean that they are upstream regulators for down-regulated genes? Also, what does this actually mean in the setting of murine VL? Can you also discuss the fact that IL-10 is up-regulated in the spleen at all time points and what it means in connection with IL-10Ra?
- Not sure what Figure 3E means, what two kinds of line means there. No description in the legend. Besides the letters are too small to see.

 

 Minor comments:

- Many abbreviations are not spelled out at their first appearance; for example, DE and GSEA are explained in full at Results section while already used in Methods. Check other abbreviations too, some are not being spelled out at all.
- Supplementary table for Figure 1A and 1B Raw data says the spleen weight of naïve mice at D36 is 412 mg. Please correct.
- Page 9: The first sentence ‘Kong et al….’ please add the reference number here too.

I have read this submission. I believe that I have an appropriate level of expertise to confirm that it is of an acceptable scientific standard.

# Response

**Published:** 2018-12-17

**Authors:** Paul Kaye

**Affiliations:** Department of Biology and Hull York Medical School, Centre for Immunology and Infection, University of York, UK

Major

*1.  comment on strain choice and specific differences in response to infection*

The reviewer is correct that we and others also use B6 mice extensively in studies of murine VL.  We have expanded in the Discussion on the rationale for our choice of BALB/c mice in this study.  However, we are currently preparing another manuscript from the CRACKIT program, which will include transcriptomic data from infected B6 as well as B6. *Rag*-/- mice.

*2. why look at a common 26 gene signature? Differences across species should be pursued.*

I think in this manuscript we have done both.  The value for a common gene signature might be in the identification of common underlying mechanisms or the identification of biomarkers that might be useful for clinical monitoring of disease progression.  We have expanded the discussion to address this. 

*3. clarify use of different time points in different species and relation to disease pattern*

Unfortunately, we are limited by the availability of data.  Researchers working with hamsters have only studied spleen (and as their study was not published at the time we began our own investigations, we could not “time match” even if that was desirable). Researchers investigating human disease have to date only published transcriptomic data from peripheral blood. We hope our current manuscript will highlight the need for better comparative studies, but it will always be difficult to match to disease profile as these differ widely between models and indeed humans. 

*4. clarify discussion on upstream negative regulators*

We have amended the text to explain how IPA determines upstream regulators

*5. improve clarity and description of Figure 3E*

We have included new text to clarify this figure.

Minor

*1. check abbreviation call outs*

These have been corrected

*2. error in Figure 1A/B RAW data table*

This has been corrected

*3. page 9: add reference to Kong et al*

This has been corrected
